# Supplementary material for: Finding New Molecular Targets of Familiar Natural Products Using In Silico Target Prediction
Source: Int J Mol Sci. 2020 Sep 26;21(19):7102. doi: 10.3390/ijms21197102 (PMC7582679; doi:10.3390/ijms21197102)
Supplement: Supplementary file 1 [file ijms-21-07102-s001.pdf]

International Journal of *Molecular Sciences*

MDPI

Article

Finding New Molecular Targets of Familiar Natural Products Using In Silico Target Prediction

Fabian Mayr [1], Gabriele Möller [2], Ulrike Garscha [3], Jana Fischer [3], Patricia Rodríguez Castaño [4,5], Silvia G. Inderbinen [6], Veronika Temml [1], Birgit Waltenberger [1], Stefan Schwaiger [1], Rolf W. Hartmann [7,8], Christian Gege [9], Stefan Martens [10], Alex Odermatt [6], Amit V. Pandey [4,5], Oliver Werz [11], Jerzy Adamski [2,12,13], Hermann Stuppner [1] and Daniela Schuster [14,15,*]

[1] Institute of Pharmacy/Pharmacognosy, Center for Molecular Biosciences Innsbruck (CMBI), University of Innsbruck, Innrain 80/82, 6020 Innsbruck, Austria
[2] Research Unit Molecular Endocrinology and Metabolism, Helmholtz Zentrum München, Ingolstädter Landstraße 1, 85764 Neuherberg, Germany
[3] Department of Pharmaceutical/Medicinal Chemistry, Institute of Pharmacy, University Greifswald, Friedrich-Ludwig-Jahn-Straße 17, 17489 Greifswald, Germany
[4] Pediatric Endocrinology, Diabetology, and Metabolism, University Children's Hospital Bern, Freiburgstrasse 15, 3010 Bern, Switzerland
[5] Department of Biomedical Research, University of Bern, Freiburgstrasse 15, 3010 Bern, Switzerland
[6] Division of Molecular and Systems Toxicology, Department of Pharmaceutical Sciences, University of Basel, Klingelbergstrasse 50, 4056 Basel, Switzerland
[7] Helmholtz Institut für Pharmazeutische Forschung, Department für Drug Design und Optimierung, Campus E8.1, 66123 Saarbrücken, Germany
[8] Universität des Saarlandes, Pharmazeutische und Medizinische Chemie, Campus E8.1, 66123 Saarbrücken
[9] Universität Heidelberg, Medizinische Chemie, Im Neuenheimer Feld 364, 69120 Heidelberg, Germany
[10] Research and Innovation Centre, Fondazione Edmund Mach (FEM), Via Mach 1, 38010 San Michele all'Adige, Italy
[11] Department of Pharmaceutical/Medicinal Chemistry, Institute of Pharmacy, Friedrich-Schiller-University Jena, Philosophenweg 14, 07743 Jena, Germany
[12] Lehrstuhl für Experimentelle Genetik, Technische Universität München, Emil-Erlenmeyer-Forum 5, 85356 Freising-Weihenstephan, Germany
[13] Department of Biochemistry, Yong Loo Lin School of Medicine, National University of Singapore, 8 Medical Drive, Singapore 117597, Singapore
[14] Institute of Pharmacy, Department of Pharmaceutical and Medicinal Chemistry, Paracelsus Medical University Salzburg, Strubergasse 21, 5020 Salzburg, Austria
[15] Institute of Pharmacy/Pharmaceutical Chemistry, Center for Molecular Biosciences Innsbruck (CMBI), University of Innsbruck, Innrain 80/82, 6020 Innsbruck, Austria
[*] Correspondence: daniela.schuster@pmu.ac.at; Tel.: +43-699-14420025 (A)

Received: date; Accepted: date; Published: date

S-1. Pharmacophore models used in this study

Table S 1. Pharmacophore Models used throughout this study.

Model Name	Software	UniProt entry	Ref.
model_DS4	DS	3BHS1_HUMAN	[1]
Model_1	LS	3BHS1_HUMAN	[1]
Model_2	LS	3BHS1_HUMAN	[1]
AChE-1acj-tha-HBA-vers	DS	ACES_HUMAN	[2]
AChE-1acj-tha-xvols	DS	ACES_HUMAN	[2]
AChE-GNT-Hypo1-shape	DS	ACES_HUMAN	[2]
AChE-X015-1	DS	ACES_HUMAN	[2]
17b-HSD5-1ry8-rut-1.60-x-1	DS	AK1C3_HUMAN	[3]
17b-HSD5-1ry8-rut-1.60-x-1-s	DS	AK1C3_HUMAN	[3]
17b-HSD5-1s2a-imn-1.70-x-1	DS	AK1C3_HUMAN	[3]
17b-HSD5-1s2a-imn-1.70-x-1-s	DS	AK1C3_HUMAN	[3]
17b-HSD5-1s2c-flf-1.80-x-1	DS	AK1C3_HUMAN	[3]
17b-HSD5-1s2c-flf-1.80-x-1-s	DS	AK1C3_HUMAN	[3]
17b-HSD5-1s2c-flf-1.80-x-2-s	DS	AK1C3_HUMAN	[3]
17b-HSD5-1zq5-e04-1.30-x-1	DS	AK1C3_HUMAN	[3]
17b-HSD5-1zq5-e04-1.30-x-1-s	DS	AK1C3_HUMAN	[3]
17b-HSD5-1zq5-e04-1.30-x-2-s	DS	AK1C3_HUMAN	[3]
17b-HSD5-2f38-15m-2.00-x-1-s	DS	AK1C3_HUMAN	[3]
17b-HSD5-X009-1	DS	AK1C3_HUMAN	[3]
17b-HSD5-X009-2	DS	AK1C3_HUMAN	[3]
FLAPophore6exclu_LS3-03	LS	AL5AP_HUMAN	[4]
FLAPophore7exclu_LS3-03	LS	AL5AP_HUMAN	[4]
LS3.12-CYP11B1_2T_D-1-8	LS	C11B1_HUMAN	[5]
LS3.12-CYP11B1_2T_D-1-8_refined-Veronika	LS	C11B1_HUMAN	[5]
LS3.12-CYP11B2_C-1-5	LS	C11B2_HUMAN	[5]
LS3.12-CYP11B2_C-1-5-refined-Veronika	LS	C11B2_HUMAN	[5]

CYP17_3RUK_LS4_03_MA	LS	CP17A_HUMAN	[1]
CYP17_Abi_Orteronel_LS4_03_MA	LS	CP17A_HUMAN	[1]
aromatase-X006-1	DS	CP19A_HUMAN	[6]
aromatase-X006-2	DS	CP19A_HUMAN	[6]
aromatase-X006-3	DS	CP19A_HUMAN	[6]
CYP2D6-quant-X012-1	DS	CP2D6_HUMAN	[7]
CYP2D6-X012-2	DS	CP2D6_HUMAN	[7]
CYP2D6-X012-3	DS	CP2D6_HUMAN	[7]
CYP2D6-X012-41	DS	CP2D6_HUMAN	[7]
CYP2D6-X012-42	DS	CP2D6_HUMAN	[7]
LS3-03b-17b-HSD2-model6-refined	LS	DHB2_HUMAN	[8]
LS3-03b-17b-HSD2-specific-model-12	LS	DHB2_HUMAN	[8]
LS3-03b-17b-HSD2-specific-model-6	LS	DHB2_HUMAN	[8]
LS3-03b-17b-HSD2-specific-model-8	LS	DHB2_HUMAN	[8]
17b-HSD3-DS-model-1-X007-1	DS	DHB3_HUMAN	[3]
17b-HSD3-DS-model-2-X007-31	DS	DHB3_HUMAN	[3]
17b-HSD3-LS3-1-317364-65-1-and-317364-71-9-model-4	LS	DHB3_HUMAN	[3]
17b-HSD3-LS3-1-317364-65-1-and-317364-71-9-model-5	LS	DHB3_HUMAN	[3]
17b-HSD3-LS3-1-875895-55-9-875895-40-2-model-1	LS	DHB3_HUMAN	[3]
17b-HSD3-LS3-1-Benzophenone-1-919118-27-7-model-1	LS	DHB3_HUMAN	[3]
17b-HSD3-LS3-1-Harada-2012-19-Fink-18m-model-4	LS	DHB3_HUMAN	[3]
17b-HSD3-LS3-1-Harada-2012-19-Fink-18m-model-7	LS	DHB3_HUMAN	[3]
17b-HSD4-quercetin-Wetzel-final	DS	DHB4_HUMAN	[3]
17b-HSD4-Quercetin-Wetzel-HBA-F-model3-2	DS	DHB4_HUMAN	[3]
17b-HSD4-Quercetin-Wetzel-HBA-F-model8-1	DS	DHB4_HUMAN	[3]
17b-HSD4-Quercetin-Wetzel-model-4-final-LS3-1	LS	DHB4_HUMAN	[3]
17b-HSD7-Fiala-DS-shapemodell_30	DS	DHB7_HUMAN	[9]
17b-HSD7-Fiala-DS-shapemodell_42	DS	DHB7_HUMAN	[9]
17b-HSD7-Fiala-DS-shapemodell_43	DS	DHB7_HUMAN	[9]
17b-HSD7-Fiala-DS-shapemodell_49	DS	DHB7_HUMAN	[9]
LS4-09-17b-HSD7-Fiala-model2-1	LS	DHB7_HUMAN	[9]

LS4-09-17b-HSD7-Fiala-model4-1	LS	DHB7_HUMAN	[9]
11b-HSD-1-refinedX005-1-HBA-model4new	DS	DHI1_HUMAN	[10]
11b-HSD1-X005-1-HBA-ohneShape-model4	DS	DHI1_HUMAN	[10]
11b-HSD1-X005-1-model1	DS	DHI1_HUMAN	[10]
11b-HSD-triterpenes-common-features	DS	DHI1_HUMAN	[10]
11b-HSD2-refinedX005-2-model2new	DS	DHI2_HUMAN	[11]
11b-HSD2-X005-2-model2	DS	DHI2_HUMAN	[11]
LS3-03a-11bHSD2-model3	LS	DHI2_HUMAN	[11]
LS3-03a11bHSD2-model5	LS	DHI2_HUMAN	[11]
LS3-03a-refined-11b-HSD2model-model3new	LS	DHI2_HUMAN	[11]
LS3-03a-refined-11bHSD2-model-model5new	LS	DHI2_HUMAN	[11]
ERa-antagonist-LS3-01-model584	LS	ESR1_HUMAN	[12]
ERa-antagonist-LS3-01-model585	LS	ESR1_HUMAN	[12]
ERa-antagonist-LS3-01-model586	LS	ESR1_HUMAN	[12]
ER-agonist-LS3-01-model009	LS	ESR1_HUMAN	[12]
ER-agonist-LS3-01-model022	LS	ESR1_HUMAN	[12]
ER-agonist-LS3-01-model030	LS	ESR1_HUMAN	[12]
ER-agonist-LS3-01-model040	LS	ESR1_HUMAN	[12]
ER-agonist-LS3-01-model042	LS	ESR1_HUMAN	[12]
ER-agonist-LS3-01-model047	LS	ESR1_HUMAN	[12]
ER-agonist-LS3-01-model052	LS	ESR1_HUMAN	[12]
ER-agonist-LS3-01-model062	LS	ESR1_HUMAN	[12]
ER-agonist-LS3-01-model069	LS	ESR1_HUMAN	[12]
ER-agonist-LS3-01-model073	LS	ESR1_HUMAN	[12]
ER-agonist-LS3-01-model084	LS	ESR1_HUMAN	[12]
ER-agonist-LS3-01-model087	LS	ESR1_HUMAN	[12]
ER-agonist-LS3-01-model099	LS	ESR1_HUMAN	[12]
ER-antagonist-LS3-01-model567	LS	ESR1_HUMAN	[12]
ER-antagonist-LS3-01-model571	LS	ESR1_HUMAN	[12]
ER-antagonist-LS3-01-model572	LS	ESR1_HUMAN	[12]
ER-antagonist-LS3-01-model573	LS	ESR1_HUMAN	[12]

ER-antagonist-LS3-01-model574	LS	ESR1_HUMAN	[12]
ER-antagonist-LS3-01-model575	LS	ESR1_HUMAN	[12]
ER-antagonist-LS3-01-model577	LS	ESR1_HUMAN	[12]
ER-antagonist-LS3-01-model578	LS	ESR1_HUMAN	[12]
ER-antagonist-LS3-01-model580	LS	ESR1_HUMAN	[12]
ER-antagonist-LS3-01-model583	LS	ESR1_HUMAN	[12]
ER-antagonist-LS3-01-model587	LS	ESR1_HUMAN	[12]
ERb-agonist-LS3-01-model100	LS	ESR2_HUMAN	[12]
ERb-agonist-LS3-01-model102	LS	ESR2_HUMAN	[12]
ERb-agonist-LS3-01-model103	LS	ESR2_HUMAN	[12]
ERb-agonist-LS3-01-model104	LS	ESR2_HUMAN	[12]
ERb-antagonist-LS3-01-model567	LS	ESR2_HUMAN	[12]
ERb-antagonist-LS3-01-model571	LS	ESR2_HUMAN	[12]
ERb-antagonist-LS3-01-model572	LS	ESR2_HUMAN	[12]
ERb-antagonist-LS3-01-model573	LS	ESR2_HUMAN	[12]
ERb-antagonist-LS3-01-model574	LS	ESR2_HUMAN	[12]
ERb-antagonist-LS3-01-model575	LS	ESR2_HUMAN	[12]
ERb-antagonist-LS3-01-model577	LS	ESR2_HUMAN	[12]
ERb-antagonist-LS3-01-model578	LS	ESR2_HUMAN	[12]
ERb-antagonist-LS3-01-model580	LS	ESR2_HUMAN	[12]
ERb-antagonist-LS3-01-model583	LS	ESR2_HUMAN	[12]
ERb-antagonist-LS3-01-model587	LS	ESR2_HUMAN	[12]
GR-1m2z-dex-2.50-T-1	DS	GCR_HUMAN	[13]
GR-1nhz-486-2.30-T-1	DS	GCR_HUMAN	[13]
GR-1nhz-486-2.30-T-2	DS	GCR_HUMAN	[13]
GR-1nhz-486-2.30-T-3	DS	GCR_HUMAN	[13]
GR-1nhz-486-2.30-T-4	DS	GCR_HUMAN	[13]
GR-1nhz-486-2.30-T-5	DS	GCR_HUMAN	[13]
GR-1p93-dex-2.70-T-1	DS	GCR_HUMAN	[13]
GR-3bqd-day-2.50-T-1	DS	GCR_HUMAN	[13]
GR-3bqd-day-2.50-T-2	DS	GCR_HUMAN	[13]

GR-3cld-gw6-2.84-T-1	DS	GCR_HUMAN	[13]
GR-3e7c-866-2.15-T-1	DS	GCR_HUMAN	[13]
GR-T001-1	DS	GCR_HUMAN	[13]
GR-T001-2	DS	GCR_HUMAN	[13]
GR-T001-3	DS	GCR_HUMAN	[13]
GR-T002-1	DS	GCR_HUMAN	[13]
GR-T002-2	DS	GCR_HUMAN	[13]
GR-T003-1	DS	GCR_HUMAN	[13]
GR-T004-1	DS	GCR_HUMAN	[13]
GR-T005-1	DS	GCR_HUMAN	[13]
GR-T006-1	DS	GCR_HUMAN	[13]
GR-T007-1	DS	GCR_HUMAN	[13]
GR-T008-1	DS	GCR_HUMAN	[13]
GR-T009-1	DS	GCR_HUMAN	[13]
GR-T010-1	DS	GCR_HUMAN	[13]
GR-T011-1	DS	GCR_HUMAN	[13]
GR-T012-1	DS	GCR_HUMAN	[13]
GR-T013-1	DS	GCR_HUMAN	[13]
GR-T014-1	DS	GCR_HUMAN	[13]
GR-T015-1	DS	GCR_HUMAN	[13]
GR-T015-2	DS	GCR_HUMAN	[13]
GR-T016-1	DS	GCR_HUMAN	[13]
GR-T017-1	DS	GCR_HUMAN	[13]
sEH-C12_1_DS3.5	DS	HYES_HUMAN	[14]
sEH-C15_1DS3.5	DS	HYES_HUMAN	[14]
sEH-1VJ5mod+3ANTmod_merged(Ref.3ANT)_LS_3-02	LS	HYES_HUMAN	[14]
sEH-1ZD5_mod5_LS_3-02	LS	HYES_HUMAN	[14]
sEH-3ANT[A]_modifiziert_LS_3-02	LS	HYES_HUMAN	[14]
sEH-3ANT+3ANTmod_merged_LS_3-02	LS	HYES_HUMAN	[14]
sEH-3I1Y_mod2_LS_3-02	LS	HYES_HUMAN	[14]
sEH-3I1Y_mod4_LS_3-02	LS	HYES_HUMAN	[14]

sEH-3KOO_mod2_LS_3-02	LS	HYES_HUMAN	[14]
sEH-3KOO_mod5_LS_3-02	LS	HYES_HUMAN	[14]
sEH-3OTQ_mod4_LS_3-02	LS	HYES_HUMAN	[14]
sEH-Cl2_Shared_mod1_LS_3-02	LS	HYES_HUMAN	[14]
5-LO-N004-1	DS	LOX5_HUMAN	[15]
5-LO-N004-2	DS	LOX5_HUMAN	[15]
5-LO-W001-5	DS	LOX5_HUMAN	[16]
LS4-09-5-LO-bzq_01v2	LS	LOX5_HUMAN	2
LS4-09-5-LO-bzq_02v2	LS	LOX5_HUMAN	2
LS4-09-5-LO-bzq_03v2	LS	LOX5_HUMAN	2
LS4-09-5-LO-bzq-02-02-16-5LOX-benzoquinone_V2	LS	LOX5_HUMAN	2
LS4-09-5-LO-zlt-20-01-16-zileuton	LS	LOX5_HUMAN	2
LS4-09-5-LO-zlt-20-01-16-zIleuton-derivatives	LS	LOX5_HUMAN	2
MR-2a3i-c0r-1.95-Z-1	DS	MCR_HUMAN	[17]
MR-2aa2-as4-1.95-Z-1	DS	MCR_HUMAN	[17]
MR-2aa2-as4-1.95-Z-2	DS	MCR_HUMAN	[17]
MR-2aa5-str-2.20-Z-1	DS	MCR_HUMAN	[17]
MR-2oax-snl-2.29-Z-1	DS	MCR_HUMAN	[17]
MR-Z001-1	DS	MCR_HUMAN	[17]
MR-Z001-2	DS	MCR_HUMAN	[17]
MR-Z002-1	DS	MCR_HUMAN	[17]
MR-Z002-2	DS	MCR_HUMAN	[17]
p38-MAPK-1a9u-sb2-800mod1_h	DS	MK14_HUMAN	[18]
p38-MAPK-1bl6-sb6-800mod1_h	DS	MK14_HUMAN	[18]
p38-MAPK-1bl7-sb4-800mod1_ha	DS	MK14_HUMAN	[18]
p38-MAPK-1bmk-sb5-800mod1_ha	DS	MK14_HUMAN	[18]
p38-MAPK-1ouk+shape	DS	MK14_HUMAN	[18]
p38-MAPK-1w82+shape	DS	MK14_HUMAN	[18]
p38-MAPK-1w83-1wbv+shape	DS	MK14_HUMAN	[18]
p38-MAPK-1wbvneu+shape	DS	MK14_HUMAN	[18]
p38-MAPK-2rg6-3cg2+shape	DS	MK14_HUMAN	[18]

p38-MAPK-3dt1-3ctq+shape	DS	MK14_HUMAN	[18]
NFkB-DNA-site-N003-1	DS	NFKB1_HUMAN	[1]
LXR-1p8d	DS	NR1H2_HUMAN	[19]
LXR-1pq6	DS	NR1H2_HUMAN	[19]
LXR-1pqc	DS	NR1H2_HUMAN	[19]
LXR-1uhl	DS	NR1H2_HUMAN	[19]
LXR-1upv	DS	NR1H2_HUMAN	[19]
LXR-1upw	DS	NR1H2_HUMAN	[19]
LXR-2acl	DS	NR1H2_HUMAN	[19]
LXR-3fal	DS	NR1H2_HUMAN	[19]
LXR-3fc6	DS	NR1H2_HUMAN	[19]
FXR-1osh-fex-1.78-d-1	DS	NR1H4_HUMAN	[20]
FXR-1osh-fex-1.78-d-1-s	DS	NR1H4_HUMAN	[20]
FXR-1osh-fex-1.78-d-2	DS	NR1H4_HUMAN	[20]
FXR-1osh-fex-1.78-d-2-s	DS	NR1H4_HUMAN	[20]
FXR-3bej-muf-1.90-x-1	DS	NR1H4_HUMAN	[20]
FXR-3bej-muf-1.90-x-1-s	DS	NR1H4_HUMAN	[20]
FXR-3bej-muf-1.90-x-2	DS	NR1H4_HUMAN	[20]
FXR-3bej-muf-1.90-x-2-s	DS	NR1H4_HUMAN	[20]
FXR-3dct-o64-2.50-x-1	DS	NR1H4_HUMAN	[20]
FXR-3dct-o64-2.50-x-1-s	DS	NR1H4_HUMAN	[20]
FXR-3dct-o64-2.50-x-2	DS	NR1H4_HUMAN	[20]
FXR-3dct-o64-2.50-x-2-s	DS	NR1H4_HUMAN	[20]
FXR-3fli-33y-2.00-x-1	DS	NR1H4_HUMAN	[20]
FXR-3fli-33y-2.00-x-1-s	DS	NR1H4_HUMAN	[20]
FXR-X008-1	DS	NR1H4_HUMAN	[20]
cPLA2alpha-N002-2	DS	PA2GA_HUMAN	[21]
COX-1-1cqe-flp-3.10-x-1	DS	PGH1_HUMAN	[22]
COX-1-1pge-isf-3.50-x-2-s	DS	PGH1_HUMAN	[22]
COX-1-2ayl-flp-2.00-x-1	DS	PGH1_HUMAN	[22]
COX-1-X017-1	DS	PGH1_HUMAN	[22]

COX-1-1EQH4_LS3-01	LS	PGH1_HUMAN	[23]
COX-1-1PGE2_LS3-01	LS	PGH1_HUMAN	[23]
COX-1-1PGG2_LS3-01	LS	PGH1_HUMAN	[23]
COX-1-2AYL3_LS3-01	LS	PGH1_HUMAN	[23]
COX-1-2OYU2_LS3-01	LS	PGH1_HUMAN	[23]
COX-2-4cox-imn-2.90-x-2	DS	PGH2_HUMAN	[22]
COX-2-6cox-s58-2.80-x-1-s	DS	PGH2_HUMAN	[22]
COX-2-3ln11_LS3-01	LS	PGH2_HUMAN	[23]
COX-2-3NTB1_LS3-01	LS	PGH2_HUMAN	[23]
COX-2-4COX2_LS3-01	LS	PGH2_HUMAN	[23]
COX-2-6COX3_LS3-01	LS	PGH2_HUMAN	[23]
PPARa-1i7g-az2-2.24-x-1	DS	PPARA_HUMAN	[24]
PPARa-1i7g-az2-2.24-x-1-s	DS	PPARA_HUMAN	[24]
PPARa-1i7g-az2-2.24-x-2	DS	PPARA_HUMAN	[24]
PPARa-1k7l-544-2.50-p-1	DS	PPARA_HUMAN	[24]
PPARa-1k7l-544-2.50-p-1-s	DS	PPARA_HUMAN	[24]
PPARa-1k7l-544-2.50-p-2	DS	PPARA_HUMAN	[24]
PPARa-1k7l-544-2.50-p-2-s	DS	PPARA_HUMAN	[24]
PPARa-1kkq-471-3.00-x-1	DS	PPARA_HUMAN	[24]
PPARa-1kkq-471-3.00-x-1-s	DS	PPARA_HUMAN	[24]
PPARa-P007-1	DS	PPARA_HUMAN	[24]
PPARd-1gwx-433-2.50-p-1	DS	PPARD_HUMAN	[24]
PPARd-1gwx-433-2.50-p-1-s	DS	PPARD_HUMAN	[24]
PPARd-1y0s-331-2.65-p-1	DS	PPARD_HUMAN	[24]
PPARd-1y0s-331-2.65-p-1-s	DS	PPARD_HUMAN	[24]
PPARd-1y0s-331-2.65-p-2-s	DS	PPARD_HUMAN	[24]
PPARd-1y0s-331-2.65-p-3	DS	PPARD_HUMAN	[24]
PPARd-1y0s-331-2.65-p-3-s	DS	PPARD_HUMAN	[24]
PPARd-2awh-vca-2.00-p-1-s	DS	PPARD_HUMAN	[24]
PPARd-2awh-vca-2.00-p-2	DS	PPARD_HUMAN	[24]
PPARd-2awh-vca-2.00-p-2-s	DS	PPARD_HUMAN	[24]

PPARd-2b50-vca-2.00-p-1-s	DS	PPARD_HUMAN	[24]
PPARd-2b50-vca-2.00-p-2	DS	PPARD_HUMAN	[24]
PPARd-2b50-vca-2.00-p-2-s	DS	PPARD_HUMAN	[24]
PPARd-2baw-vca-2.30-p-1-s	DS	PPARD_HUMAN	[24]
PPARd-2j14-gni-2.80-p-1-s	DS	PPARD_HUMAN	[24]
PPARd-3gwx-epa-2.40-p-1-s	DS	PPARD_HUMAN	[24]
PPARd-3gwx-epa-2.40-p-2-s	DS	PPARD_HUMAN	[24]
PPARg-1fm6-brl-2.10-p-1	DS	PPARG_HUMAN	[24]
PPARg-1fm6-brl-2.10-p-1-s	DS	PPARG_HUMAN	[24]
PPARg-1fm9-570-2.10-x-1	DS	PPARG_HUMAN	[24]
PPARg-1fm9-570-2.10-x-1-s	DS	PPARG_HUMAN	[24]
PPARg-1fm9-570-2.10-x-2	DS	PPARG_HUMAN	[24]
PPARg-1fm9-570-2.10-x-2-s	DS	PPARG_HUMAN	[24]
PPARg-1i7i-az2-2.35-x-1	DS	PPARG_HUMAN	[24]
PPARg-1i7i-az2-2.35-x-1-s	DS	PPARG_HUMAN	[24]
PPARg-1k74-544-2.30-p-1-s	DS	PPARG_HUMAN	[24]
PPARg-1knu-ypa-2.50-p-1-s	DS	PPARG_HUMAN	[24]
PPARg-1knu-ypa-2.50-p-2-s	DS	PPARG_HUMAN	[24]
PPARg-1nyx-drf-2.65-p-1	DS	PPARG_HUMAN	[24]
PPARg-1nyx-drf-2.65-p-1-s	DS	PPARG_HUMAN	[24]
PPARg-1nyx-drf-2.65-p-2-s	DS	PPARG_HUMAN	[24]
PPARg-1wm0-plb-2.90-p-1-s	DS	PPARG_HUMAN	[24]
PPARg-1zeo-c01-2.50-p-1	DS	PPARG_HUMAN	[24]
PPARg-1zeo-c01-2.50-p-1-s	DS	PPARG_HUMAN	[24]
PPARg-1zgy-brl-1.80-p-1-s	DS	PPARG_HUMAN	[24]
PPARg-2ath-3ea-2.28-p-1-s	DS	PPARG_HUMAN	[24]
PPARg-2f4b-eha-2.07-p-1-s	DS	PPARG_HUMAN	[24]
PPARg-2fvj-ro0-1.99-p-1-s	DS	PPARG_HUMAN	[24]
PPARg-2g0g-sp0-2.54-p-1	DS	PPARG_HUMAN	[24]
PPARg-2g0g-sp0-2.54-p-1-s	DS	PPARG_HUMAN	[24]
PPARg-2g0h-sp3-2.30-p-1-s	DS	PPARG_HUMAN	[24]

PPARg-2gtk-208-2.10-p-1-s	DS	PPARG_HUMAN	[24]
PPARg-2hfp-nsi-2.00-p-1-s	DS	PPARG_HUMAN	[24]
PPARg-2prg-brl-2.30-p-1	DS	PPARG_HUMAN	[24]
PPARg-2prg-brl-2.30-p-1-s	DS	PPARG_HUMAN	[24]
PPARg-2q59-240-2.20-p-1-s	DS	PPARG_HUMAN	[24]
PPARg-4prg-072-2.90-p-1-s	DS	PPARG_HUMAN	[24]
PPARg-P002-1	DS	PPARG_HUMAN	[24]
mPGES-1-Hypo_62_01-non-acidic	DS	PTGES_HUMAN	[25]
mPGES-1-X019-1	DS	PTGES_HUMAN	[25]
mPGES-1-X019-2	DS	PTGES_HUMAN	[25]
mPGES-1-model-LS3-01	LS	PTGES_HUMAN	[25]
PTP1b-1bzc-tpi-2.35-d-2	DS	PTN1_HUMAN	[26]
PTP1b-1bzh-flt-2.10-d-1	DS	PTN1_HUMAN	[26]
PTP1b-1bzj-pic-2.25-d-1	DS	PTN1_HUMAN	[26]
PTP1b-1c83-oai-1.80-d-1-s	DS	PTN1_HUMAN	[26]
PTP1b-1c83-oai-1.80-d-2	DS	PTN1_HUMAN	[26]
PTP1b-1c83-oai-1.80-d-2-s	DS	PTN1_HUMAN	[26]
PTP1b-1c84-761-2.35-d-1	DS	PTN1_HUMAN	[26]
PTP1b-1c84-761-2.35-d-1-s	DS	PTN1_HUMAN	[26]
PTP1b-1c84-761-2.35-d-2	DS	PTN1_HUMAN	[26]
PTP1b-1c84-761-2.35-d-2-s	DS	PTN1_HUMAN	[26]
PTP1b-1c84-761-2.35-d-3	DS	PTN1_HUMAN	[26]
PTP1b-1c84-761-2.35-d-3-s	DS	PTN1_HUMAN	[26]
PTP1b-1c85-oba-2.72-d-1	DS	PTN1_HUMAN	[26]
PTP1b-1c85-oba-2.72-d-1-s	DS	PTN1_HUMAN	[26]
PTP1b-1c86-opa-2.30-d-1	DS	PTN1_HUMAN	[26]
PTP1b-1c86-opa-2.30-d-1-s	DS	PTN1_HUMAN	[26]
PTP1b-1c87-opa-2.10-d-1	DS	PTN1_HUMAN	[26]
PTP1b-1c87-opa-2.10-d-1-s	DS	PTN1_HUMAN	[26]
PTP1b-1c88-ota-1.80-d-1	DS	PTN1_HUMAN	[26]
PTP1b-1ecv-878-1.95-d-1	DS	PTN1_HUMAN	[26]

PTP1b-1ecv-878-1.95-d-1-s	DS	PTN1_HUMAN	[26]
PTP1b-1ecv-878-1.95-d-2	DS	PTN1_HUMAN	[26]
PTP1b-1ecv-878-1.95-d-2-s	DS	PTN1_HUMAN	[26]
PTP1b-1ecv-878-1.95-d-3	DS	PTN1_HUMAN	[26]
PTP1b-1ecv-878-1.95-d-3-s	DS	PTN1_HUMAN	[26]
PTP1b-1g7g-inx-2.20-d-1	DS	PTN1_HUMAN	[26]
PTP1b-1gfy-col-2.13-d-1	DS	PTN1_HUMAN	[26]
PTP1b-1gfy-col-2.13-d-1-s	DS	PTN1_HUMAN	[26]
PTP1b-1jf7-tbh-2.20-d-1	DS	PTN1_HUMAN	[26]
PTP1b-1kak-fnp-2.50-d-1	DS	PTN1_HUMAN	[26]
PTP1b-1kak-fnp-2.50-d-2	DS	PTN1_HUMAN	[26]
PTP1b-1l8g-dbd-2.50-d-1	DS	PTN1_HUMAN	[26]
PTP1b-1nl9-989-2.40-d-1	DS	PTN1_HUMAN	[26]
PTP1b-1nl9-989-2.40-d-1-s	DS	PTN1_HUMAN	[26]
PTP1b-1nny-515-2.40-d-1	DS	PTN1_HUMAN	[26]
PTP1b-1no6-794-2.40-d-1	DS	PTN1_HUMAN	[26]
PTP1b-1no6-794-2.40-d-1-s	DS	PTN1_HUMAN	[26]
PTP1b-1nwl-964-2.40-d-1	DS	PTN1_HUMAN	[26]
PTP1b-1nwl-964-2.40-d-1-s	DS	PTN1_HUMAN	[26]
PTP1b-1nz7-901-2.40-d-1	DS	PTN1_HUMAN	[26]
PTP1b-1ony-588-2.15-d-1	DS	PTN1_HUMAN	[26]
PTP1b-1ony-588-2.15-d-1-s	DS	PTN1_HUMAN	[26]
PTP1b-1onz-968-2.40-d-1	DS	PTN1_HUMAN	[26]
PTP1b-1onz-968-2.40-d-1-s	DS	PTN1_HUMAN	[26]
PTP1b-1ph0-418-2.20-d-1	DS	PTN1_HUMAN	[26]
PTP1b-1ph0-418-2.20-d-2	DS	PTN1_HUMAN	[26]
PTP1b-1ph0-418-2.20-d-2-s	DS	PTN1_HUMAN	[26]
PTP1b-1pxh-sna-2.15-d-1	DS	PTN1_HUMAN	[26]
PTP1b-1pyn-941-2.20-d-1	DS	PTN1_HUMAN	[26]
PTP1b-1q1m-234-2.60-d-1	DS	PTN1_HUMAN	[26]
PTP1b-1q6j-335-2.20-d-1	DS	PTN1_HUMAN	[26]

PTP1b-1q6j-335-2.20-d-1-s	DS	PTN1_HUMAN	[26]
PTP1b-1q6m-p27-2.20-d-1	DS	PTN1_HUMAN	[26]
PTP1b-1q6m-p27-2.20-d-1-s	DS	PTN1_HUMAN	[26]
PTP1b-1q6n-p90-2.10-d-1	DS	PTN1_HUMAN	[26]
PTP1b-1q6n-p90-2.10-d-1-s	DS	PTN1_HUMAN	[26]
PTP1b-1q6p-213-2.30-d-1	DS	PTN1_HUMAN	[26]
PTP1b-1q6s-214-2.20-d-1	DS	PTN1_HUMAN	[26]
PTP1b-1q6s-214-2.20-d-1-s	DS	PTN1_HUMAN	[26]
PTP1b-1q6t-600-2.30-d-1	DS	PTN1_HUMAN	[26]
PTP1b-1qxk-429-2.30-d-1	DS	PTN1_HUMAN	[26]
PTP1b-2bgd-t1d-2.40-d-1	DS	PTN1_HUMAN	[26]
PTP1b-2cne-dfj-1.80-d-1	DS	PTN1_HUMAN	[26]
PTP1b-2cne-dfj-1.80-d-1-s	DS	PTN1_HUMAN	[26]
PTP1b-2cne-dfj-1.80-d-2	DS	PTN1_HUMAN	[26]
PTP1b-2cne-dfj-1.80-d-2-s	DS	PTN1_HUMAN	[26]
PTP1b-2cnf-f32-2.20-d-1	DS	PTN1_HUMAN	[26]
PTP1b-2cnf-f32-2.20-d-1-s	DS	PTN1_HUMAN	[26]
PTP1b-2cnf-f32-2.20-d-2	DS	PTN1_HUMAN	[26]
PTP1b-2cnf-f32-2.20-d-2-s	DS	PTN1_HUMAN	[26]
PTP1b-2cng-ize-1.90-d-1	DS	PTN1_HUMAN	[26]
PTP1b-2cng-ize-1.90-d-1-s	DS	PTN1_HUMAN	[26]
PTP1b-2cnh-izb-1.80-d-1	DS	PTN1_HUMAN	[26]
PTP1b-2cnh-izb-1.80-d-1-s	DS	PTN1_HUMAN	[26]
PTP1b-2cni-izf-2.00-d-1	DS	PTN1_HUMAN	[26]
PTP1b-2cni-izf-2.00-d-1-s	DS	PTN1_HUMAN	[26]
PTP2b-1g7f-inz-1.80-d-1	DS	PTN1_HUMAN	[26]
LS3-03b-1C85-1-model	LS	PTN1_HUMAN	[26]
LS3-03b-1C85-2-model	LS	PTN1_HUMAN	[26]
LS3-03b-1PH0-model	LS	PTN1_HUMAN	[26]
LS3-03b-1PYN-model	LS	PTN1_HUMAN	[26]
LS3-03b-1Q6S-model	LS	PTN1_HUMAN	[26]

LS3-03b-1T49-model	LS	PTN1_HUMAN	[26]
LS3-03b-2CM7-model	LS	PTN1_HUMAN	[26]
LS3-03b-2CNG-model	LS	PTN1_HUMAN	[26]
LS3-03b-2F71-model	LS	PTN1_HUMAN	[26]
LS3-03b-2QBS-model	LS	PTN1_HUMAN	[26]
IKK2-3rzf-xnm-4.00-n-1	DS	Q6INT1_XENLA	[27]
IKK2-N001-6	DS	Q6INT1_XENLA	[27]
5a-red-R003-2	DS	S5A2_HUMAN	[28]
5a-red-R003-4	DS	S5A2_HUMAN	[28]
STS-LS3.12-model10-mostafa-2012a-21-maltais-2009a-55-XVols-binding-site	LS	STS_HUMAN	[29]
STS-LS3.12-model11-lehr-2005a-4f-nussbaumer-2003b-6g	LS	STS_HUMAN	[29]
STS-LS3.12-model12-mostafa-2012a-21-maltais-2009a-55-XVols-binding-site-HBD	LS	STS_HUMAN	[29]

[1] not published. [2] in preparation.

S-2. Supplementary Figures

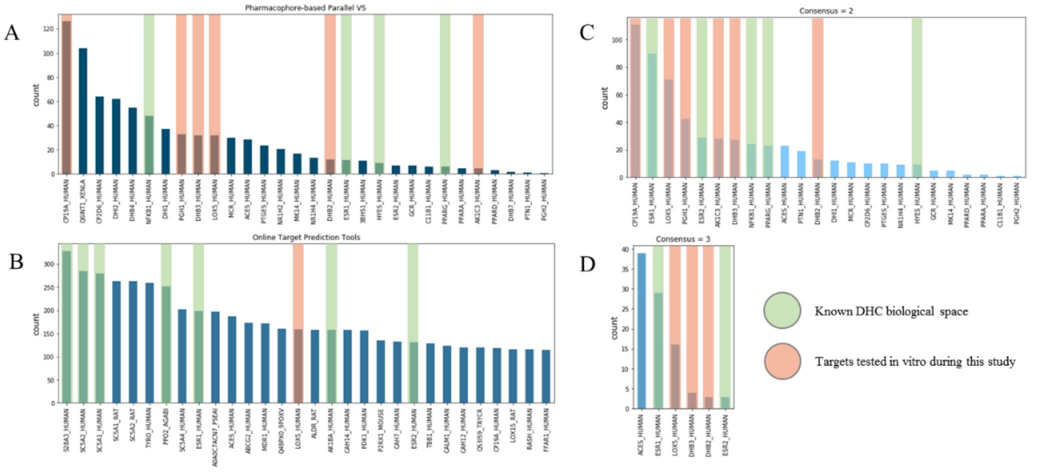

3BHS1_HUMAN	3β hydroxysteroid dehydrogenase 1	*DHB2_HUMAN*	17β HSD2	*MK14_HUMAN*	MAP kinase 14	*Q49PX0_9POXV*	N1L
A0A0C7ACN7_P.	3-oxoacyl-ACP synthase	*DHB3_HUMAN*	17β HSD3	*NFKB1_HUMAN*	NF κB p105 subunit	*Q53I59_TRYCR*	Phosphodiesterase
ABCG2_HUMAN	ATP-binding cassette G2	*DHB4_HUMAN*	17β HSD4	*NR1H2_HUMAN*	Oxysterols receptor β	*Q6INT1_XENLA*	MGC80376 protein
ACES_HUMAN	Acetylcholinesterase	*DHB7_HUMAN*	3-keto-steroid reductase	*NR1H4_HUMAN*	Bile acid receptor	*RASH_HUMAN*	GTPase HRas
AK1BA_HUMAN	Aldo-keto reductase 1B10	*DHI1_HUMAN*	11β HSD1	*P2RX1_MOUSE*	P2X purinoceptor 1	*S28A3_HUMAN*	Solute carrier 28A3
AK1C3_HUMAN	Aldo-keto reductase 1C3	*ESR1_HUMAN*	Estrogen receptor α	*PDK1_HUMAN*	Pyruvate dehydrogenase	*SC5A1_HUMAN*	Sodium/glucose cotransporter 1
ALDR_RAT	Aldo-keto reductase 1B1 (rat)	*ESR2_HUMAN*	Estrogen receptor β	*PGH1_HUMAN*	COX-1	*SC5A1_RAT*	Sodium/glucose cotransporter 1
C11B1_HUMAN	Cytochrome P450 11B1	*FFAR1_HUMAN*	Free fatty acid receptor 1	*PGH2_HUMAN*	COX-2	*SC5A2_HUMAN*	Sodium/glucose cotransporter 2
CAH12_HUMAN	Carbonic anhydrase 12	*GCR_HUMAN*	Glucocorticoid receptor	*PPARA_HUMAN*	PPAR α	*SC5A2_RAT*	Sodium/glucose cotransporter 2
CAH14_HUMAN	Carbonic anhydrase 14	*HYES_HUMAN*	Soluble epoxide hydrolase 2	*PPARD_HUMAN*	PPAR δ	*SC5A4_HUMAN*	Solute carrier family 5 member 4
CAH7_HUMAN	Carbonic anhydrase 7	*LOX15_RAT*	15-lipoxygenase (rat)	*PPARG_HUMAN*	PPAR γ	*TBB1_HUMAN*	Tubulin β chain
CALM1_HUMAN	Calmodulin-1	*LOX5_HUMAN*	5-lipoxygenase	*PPO2_AGABI*	Mushroom tyrosinase	*TYRO_HUMAN*	Tyrosinase
CP19A_HUMAN	Aromatase	*MCR_HUMAN*	Mineralocorticoid receptor	*PTGES_HUMAN*	Prostaglandin E synthase		
CP2D6_HUMAN	Cytochrome P450 2D6	*MDR1_HUMAN*	ATP-dependent translocase ABCB1	*PTN1_HUMAN*	PTP1B		

Figure S 1. Enrichment of known DHC targets (known DHC biological space) in differently scored predictions for DHC chemical space. (A) Predictions made with stand-alone Ph-DB. (B) Predictions made with SEA, STP, and SP combined, and all four approaches combined with the consensus score of two (C) and three applied (D). Previously known DHC targets are highlighted with green, and targets selected for in vitro evaluation during this study with red overlays.

Metrics Computed for the Isolated and Combined Target Prediction Tools

	Metric	AKR1C3	17β HSD3	17β HSD2	Aromatase	5-LO	COX-1	Mean
**Consenensus Score (CS)**	Recall	1.00	0	0	0	0.86	0.75	0.44
	Precision	0.67	0	0	0	0.86	0.50	0.34
	Relative EF	0.67	0	0	0	0.86	0.50	0.34
**pharmacophore-based parallel VS (Ph-DB)**	Recall	0	0	0	0	0.57	0.50	0.18
	Precision	0	0	0	0	0.80	0.50	0.22
	Relative EF	0	0	0	0	0.80	0.50	0.22
**Similarity Ensemble Approach (SEA)**	Recall	1.00	0	0	0	0.29	0	0.22
	Precision	0.67	0	0	0	0.67	0	0.22
	Relative EF	0.67	0	0	0	0.67	0	0.22
**SwissTarget Prediction (STP)**	Recall	0	0	0	0	0.29	0.25	0.27
	Precision	0	0	0	0	0.67	0.50	0.20
	Relative EF	0	0	0	0	0.67	0.50	0.20
**SuperPred (SP)**	Recall	0	0	0	0	0	0	0
	Precision	0	0	0	0	0	0	0
	Relative EF	0	0	0	0	0	0	0

True/False Positives/Negatives

True Positives (TPs) refer to actives that were predicted as active, while False Positives (FPs) refer to inactives that were predicted as active. Vice versa, True Negatives (TNs) are inactives predicted as active, while False Negatives (FNs) are actives predicted as inactive.

Recall/Sensitivity

Proportion of true actives a model is able to retrieve from the screening dataset (0 - 1).

$$recall = \frac{TP}{TP + FN}$$

Precision

Proportion of true actives in a hitlist produced by a model (0 - 1).

$$precision = \frac{TP}{TP + FP}$$

Relative Enrichment Factor

Ability of a model to enrich a hitlist with true positive predictions (0 - 1).

$$relative\ EF = \frac{\frac{TP}{TP+FP}}{\frac{actives}{inactives}} * \frac{1}{\frac{actives}{inactives}}$$

Figure S 2. Metrics computed for the isolated and combined target prediction tools.

A

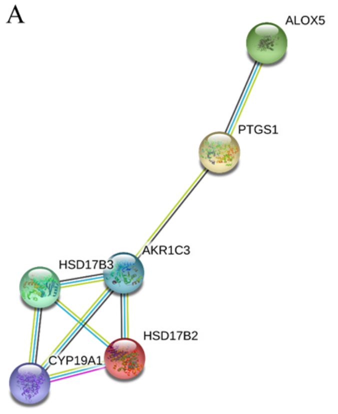

B

Node 1		Node 2		STRING Score
**STRING name**	**alternative name**	**STRING name**	**alternative name**	
AKR1C3	AKR1C3	CYP19A1	aromatase	0.952
AKR1C3	AKR1C3	HSD17B2	17β HSD2	0.924
AKR1C3	AKR1C3	HSD17B3	17β HSD3	0.935
AKR1C3	AKR1C3	PTGS1	COX-1	0.452
ALOX5	5-LO	PTGS1	COX-1	0.973
CYP19A1	aromatase	AKR1C3	AKR1C3	0.952
CYP19A1	aromatase	HSD17B2	17β HSD2	0.962
CYP19A1	aromatase	HSD17B3	17β HSD3	0.958
HSD17B2	17β HSD2	AKR1C3	AKR1C3	0.924
HSD17B2	17β HSD2	CYP19A1	aromatase	0.962
HSD17B2	17β HSD2	HSD17B3	17β HSD3	0.979
HSD17B3	17β HSD3	AKR1C3	AKR1C3	0.935
HSD17B3	17β HSD3	CYP19A1	aromatase	0.958
HSD17B3	17β HSD3	HSD17B2	17β HSD2	0.979
PTGS1	COX-1	AKR1C3	AKR1C3	0.452
PTGS1	COX-1	ALOX5	5-LO	0.973

C

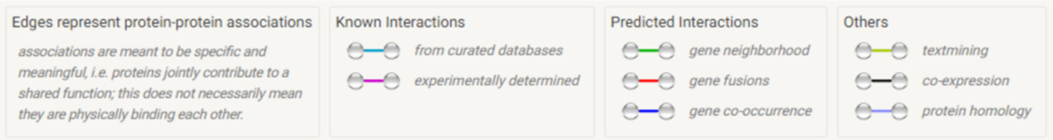

Figure S 3. Six potential protein targets for DHCs input to STRING, which creates a network of both direct and functional protein-protein interactions [30].

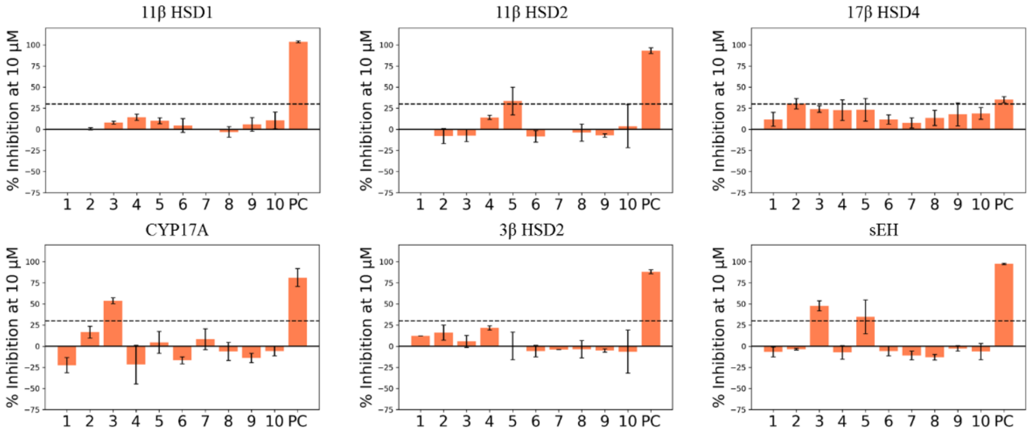

Figure S 4. Results of other protein targets tested in the course of this study due to assay availability shown as bar charts. Compounds **1** – **10** indicated with the respective means (n = 3) of percent inhibition at 10 µM (0 – 100%) and standard deviation. Black dashed line again indicates arbitrarily chosen 30% activity cut-off. DMSO was used to measure baseline enzyme activities, on which samples were normalized (not shown) and positive controls (PC) were used as indicated in Materials and Methods.

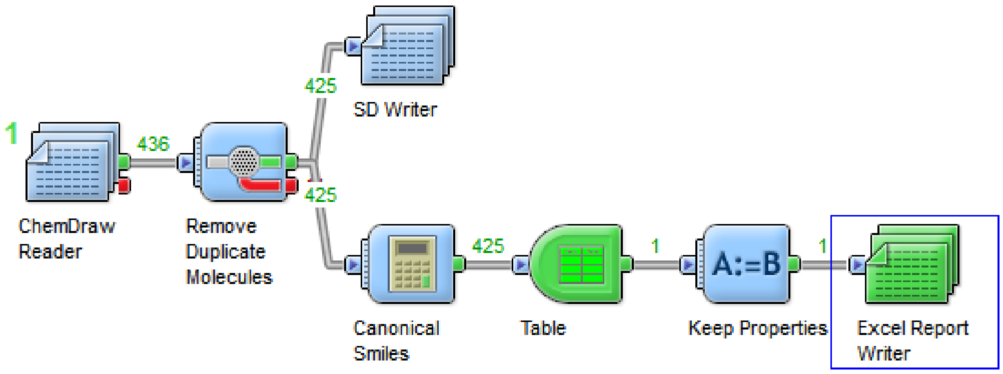

Figure S 5. Pipeline Pilot protocol '*chemdraw_to_sd_smiles_fabian.xml*' used to convert ChemDraw's native file format '*.cdx*' to an '*.sdf*'-file and a '*.csv*' table with isomeric smiles codes while maintaining issued stereochemistry. Removal of duplicate entries is necessary, since various names of the compounds can be found in literature, and consequently in the hand-drawn library. The protocol is further provided on GitHub (https://github.com/fmayr/DHC_TargetPrediction).

Table S 2. Parameter settings used for the Ligand Profiler protocol used in parallel VS with Discovery Studio.

**Protocol Settings**	**Protocol.pr_xml**	
	Input Ligands	DHC_full.sdf
	Input LigandProfilerDB Pharmacophores	
	Input PharmaDB Pharmacophores	
Model Selection	Most Selective	
	Conformation Generation	BEST
Maximum Conformations	255	
Discard Existing Conformations	WAHR	
Energy Threshold	20	
Ring Fragments File		
Save Conformations	FALSCH	
	Advanced	
Input Type	Ligands	
Input Database	Sample	
Input Database Limit Hits	First N	
Input Database Maximum	300	
Input Database Hitlist		
Fitting Method	Rigid	
Maximum Omitted Features	0	
Minimum Interfeature Distance	0.00001	
Scale Fit Values	WAHR	
Prune Empty Fits	WAHR	
Prune Missed Molecules	WAHR	
Keep Input Conformations	FALSCH	
Save Aligned Ligands	FALSCH	

Activity Property		
Catalyst Parameter File		
	Parallel Processing	FALSCH

S-3. File Scheme

Table S 3. Files used and produced during this study. Every file is freely available at GitHub (https://github.com/fmayr/DHC_TargetPrediction). For greater clarity, a file scheme is provided in Supplementary Information S-2 describing all dependencies.

File Name	Contains	Subfolder
DHC_full.csv	DHC chemical space as csv-file (name, smiles).	/dataset
DHC_full.sdf	DHC chemical space as 3D-molecule files.	/dataset
DHC_full_lit_network.csv	Known DHC biological space and result of bioactivity mining. Ready to be imported to Cytoscape.	/bioactivity%20mining
DHC_full_online.csv	Result produced by online target prediction servers (SEA, STP, SP) and DHC_full.csv as input.	/TarPredCrawler
DHC_full_LS_mergedhits.csv	Csv-file of hitlists produced by LigandScout models in Ph-DB.	/pharmacophore-based parallel VS
DHC_full_ligandprofiler.csv	Csv-file of hitlists produced by Discovery Studio model in Ph-DB.	/pharmacophore-based parallel VS
DHC_full_inhouse.csv	Joined results of LigandScout and Discovery Studio outputs.	/pharmacophore-based parallel VS
DHC_full_pivoted.csv	Joined results of SEA, STP, SP, and Ph-DB predictions for DHC chemical space.	
DHC_10_pivoted.csv	DHC_full_pivoted.csv filtered for compounds 1 – 10.	
DHC_10_network.csv	DHC_10_pivoted.csv joined with DHC_full_lit_network.csv. Network file ready to be imported to Cytoscape. Contains known and predicted compound-target associations.	
Bioactivity_network_generator_SMILES.py	Python script used for literature mining. For installation instruction see README.	
TarPredCrawler.py	Python script used for submitting and collecting results from SEA, STP, and SP.	
DHC_targetpreidction_datatreatment.ipynb	Jupyter Notebook containing all data treatment and plotting performed in this study.	

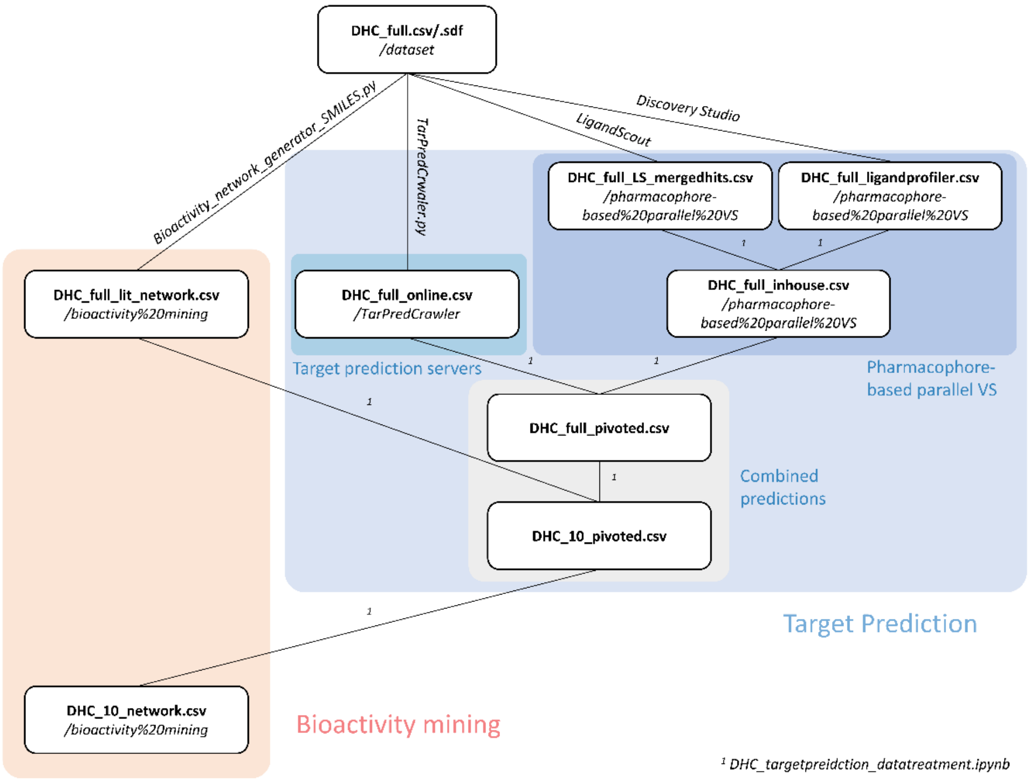

Figure S 6. File scheme describing the course of this study. All files are available for download on GitHub (https://github.com/fmayr/DHC_TargetPrediction), relative paths are written in italics.

S-4. Quality Control of Compounds 1-10

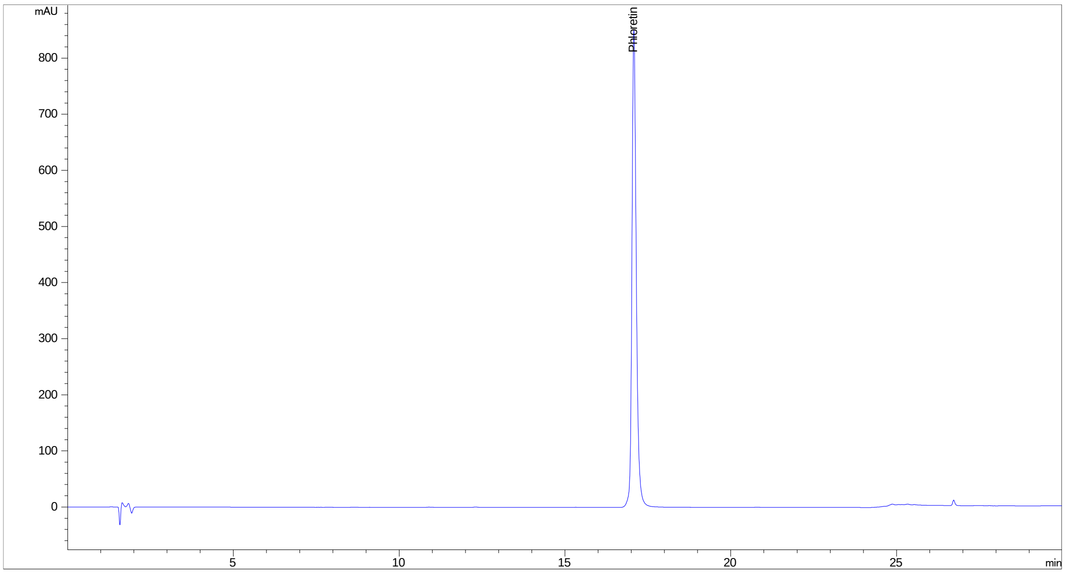

Figure S 7. Phloretin (1) CAS: 60-82-2

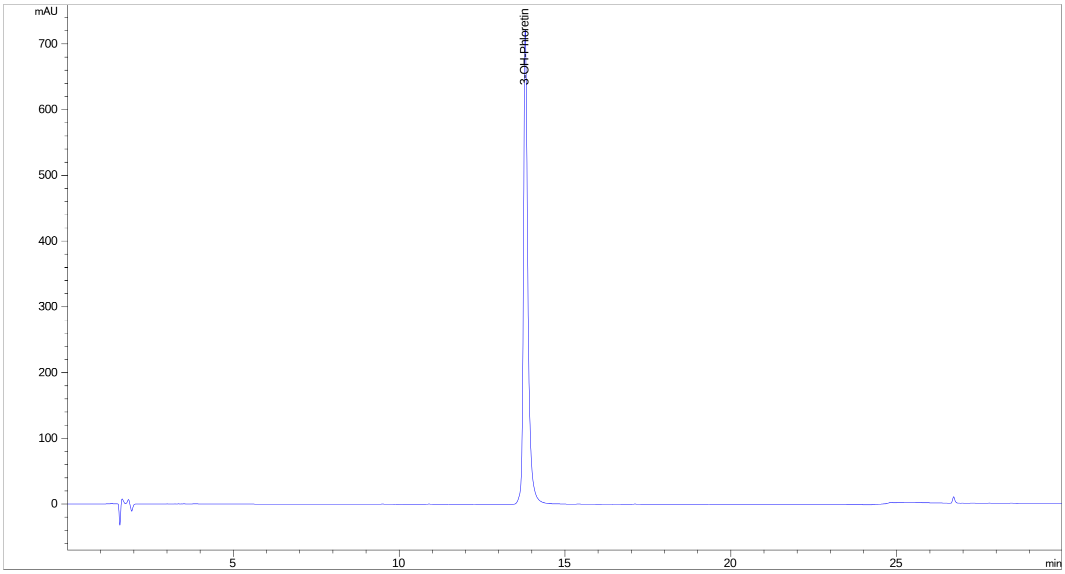

Figure S 8. 3-OH-phloretin (2) CAS: 57765-66-9

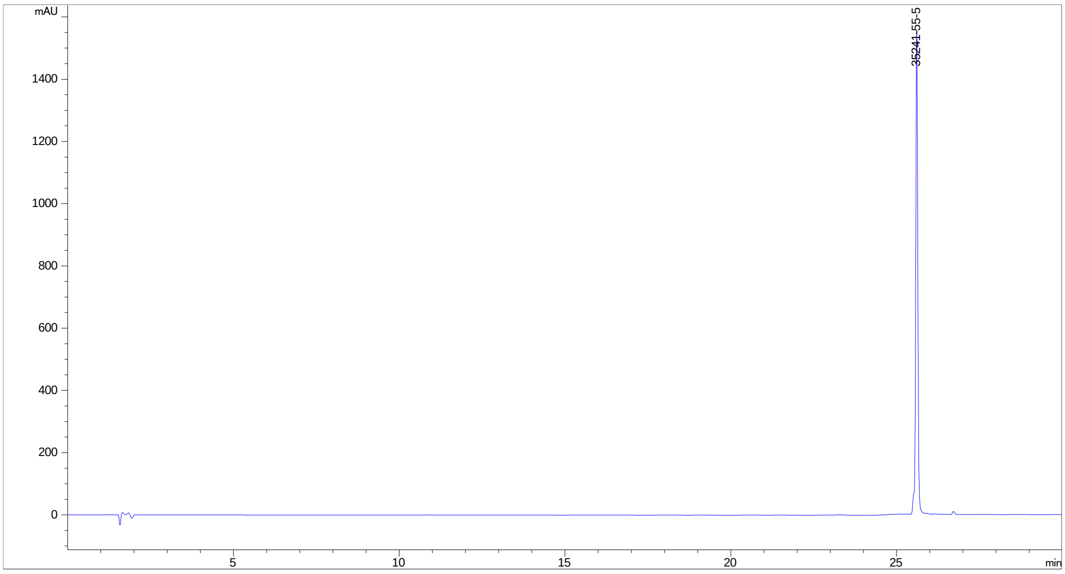

Figure S 9. 2',6'-dihydroxy-4'-methoxy DHC (3) CAS: 35241-55-5

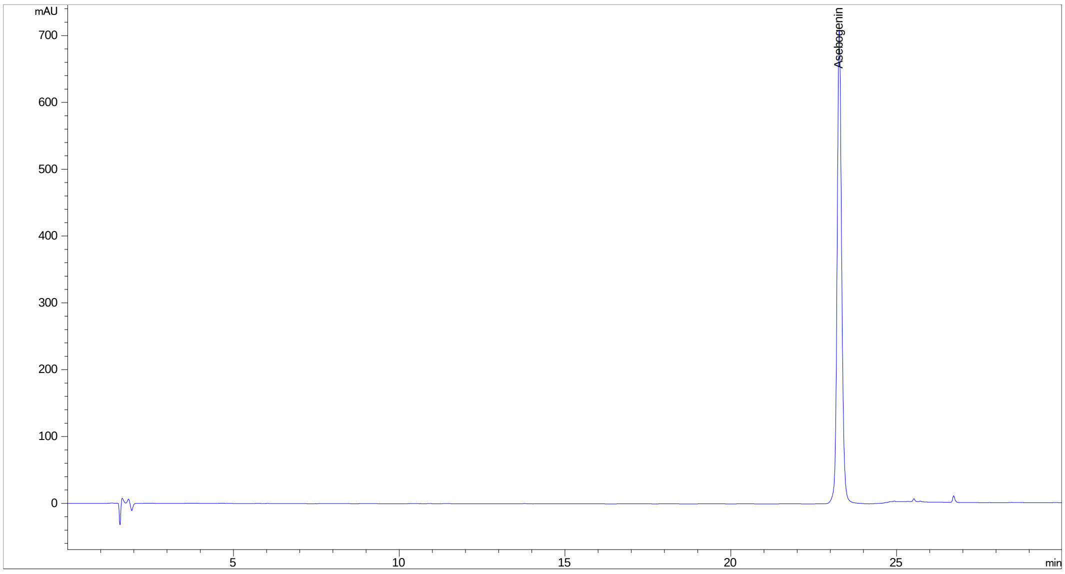

Figure S 10. Asebogenin (4) CAS: 35241-54-4

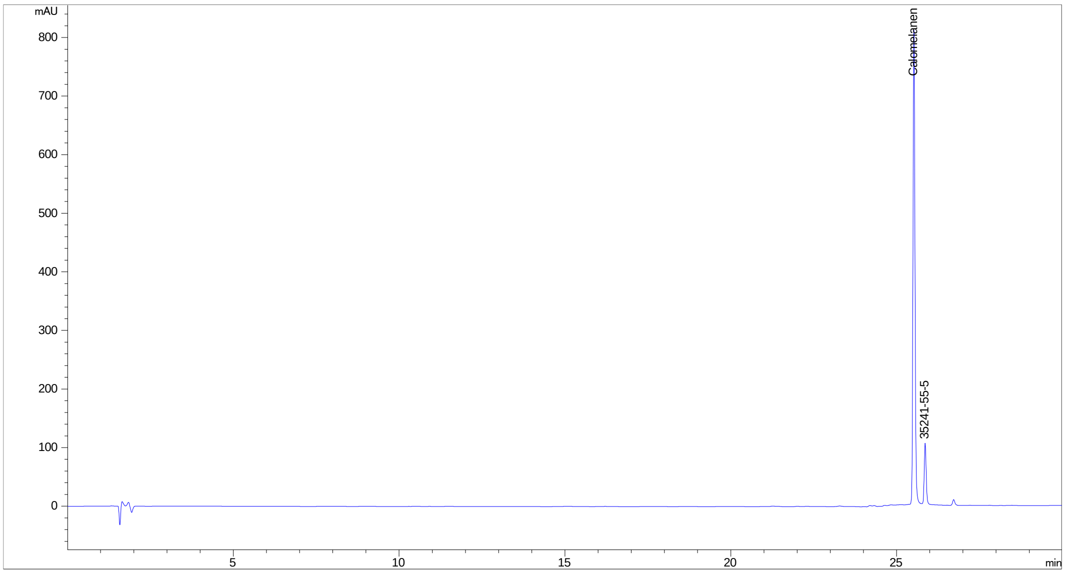

Figure S 11. Calomelanen (5) CAS: 520-42-3

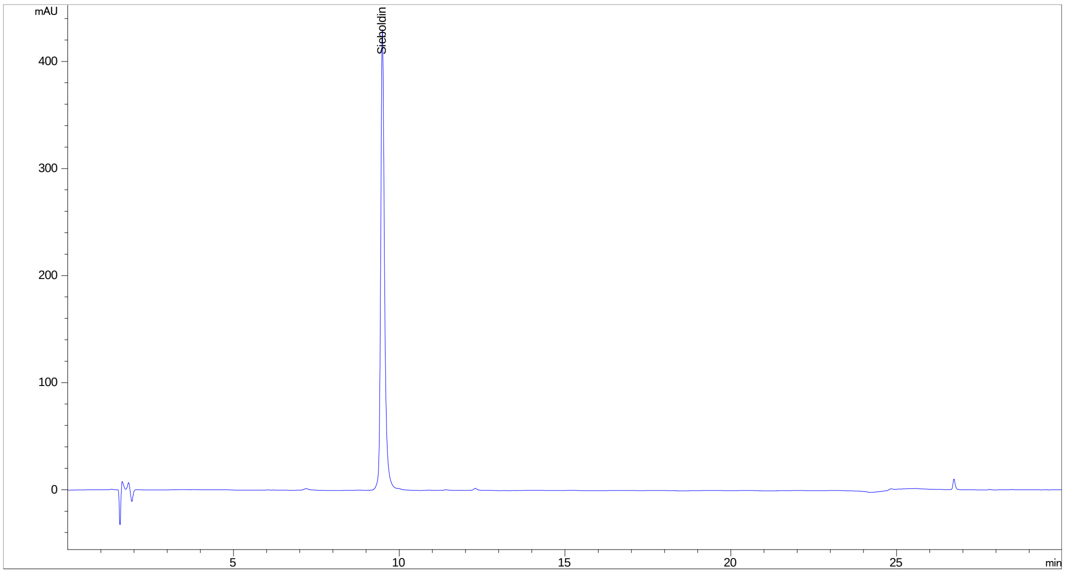

Figure S 12. Sieboldin (6) CAS: 18777-73-6

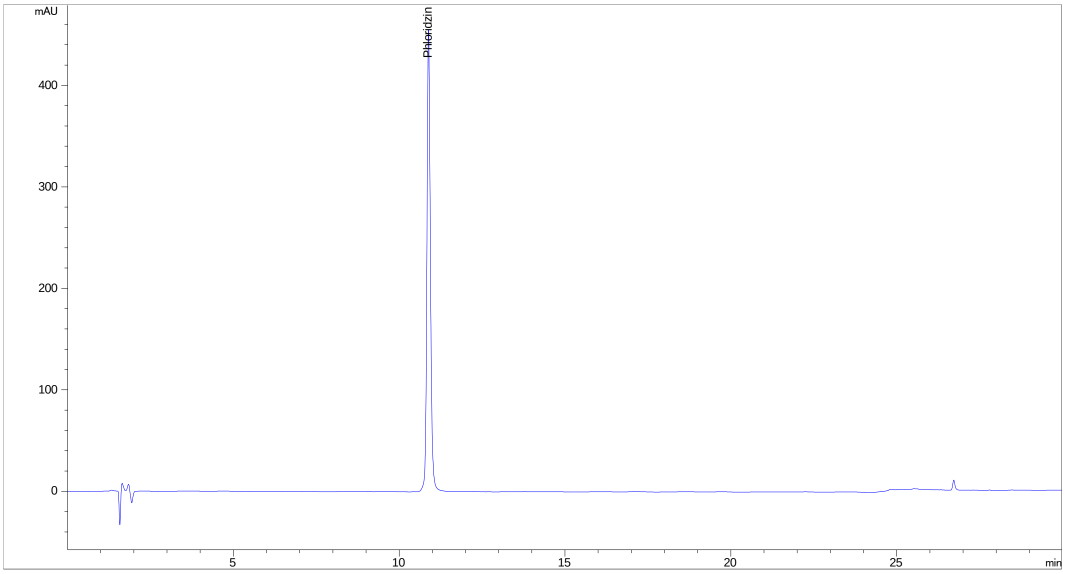

Figure S 13. Phloridzin (7) CAS: 60-81-1

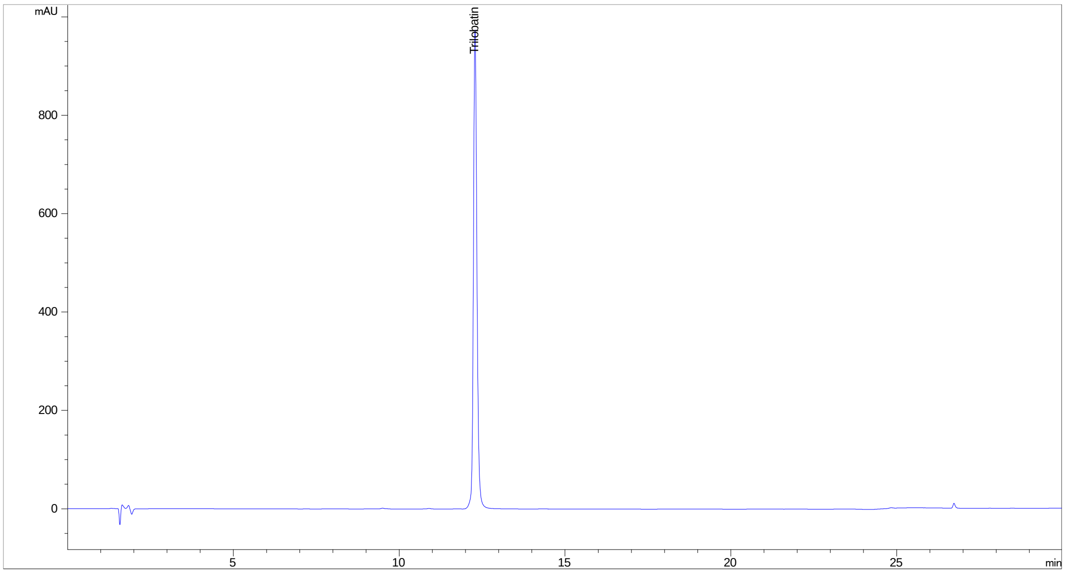

Figure S 14. Trilobatin (8) CAS: 4192-90-9.

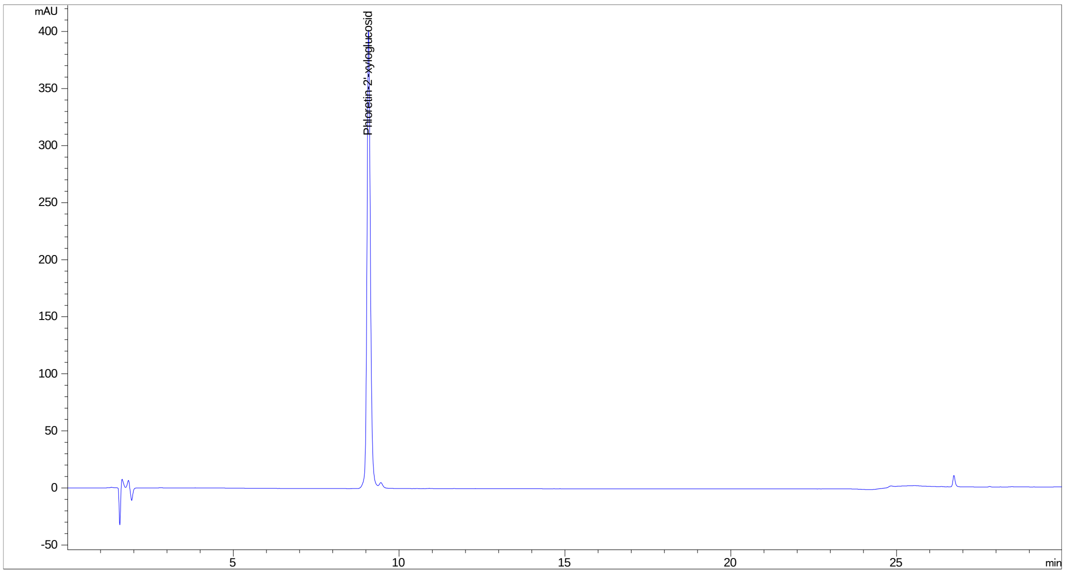

Figure S 15. Phloretin-2'-xyloglucoside (9) CAS: 145758-09-4.

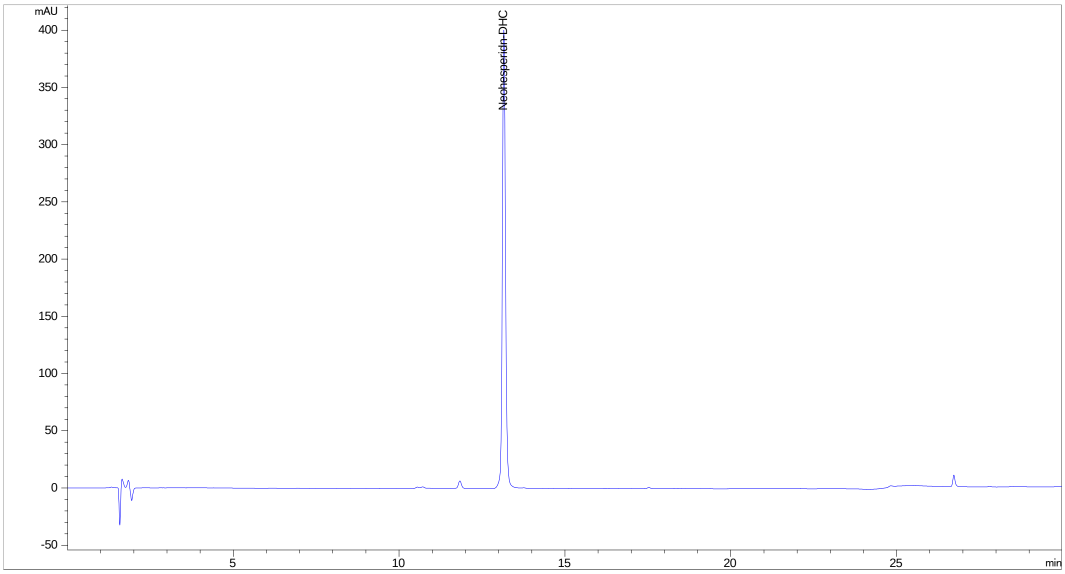

Figure S 16. Neohesperidin DHC (10) CAS: 20702-77-6.

S-5. Positive Controls

Table S 4. Positive controls used during this study.

Assay	Name	CAS	Structure	Ref.
**3β HSD1**	Trilostane	13647-35-3	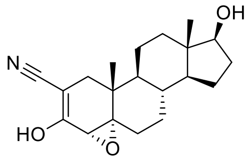	[31,32]
**11β HSD1**<br>**11β HSD2**	18β-Glycyrrhetinic acid	471-53-4	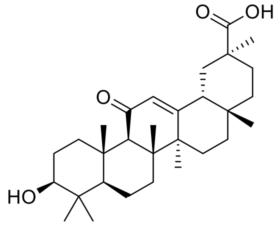	[33]
**17β HSD2**<br>**17β HSD4**	compound 19	1340482-23-6	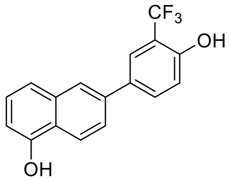	[34]
**17β HSD3**	compound 24	873206-61-2	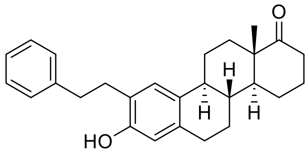	[35]
**5-LO**	Zileuton	111406-87-2	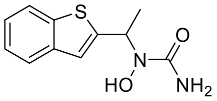	[36]
**aromatase**	anastrozole	120511-73-1	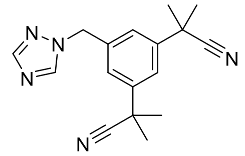	[37]
**AKR1C3**	compound 2-9	745028-76-6	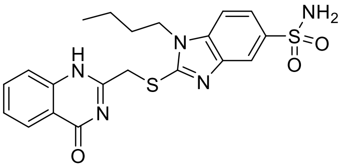	[3]
**COX-1**	Indomethacin	53-86-1	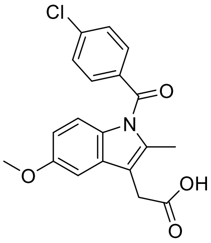	[38]
**CYP17A1**	Abiraterone	154229-19-3	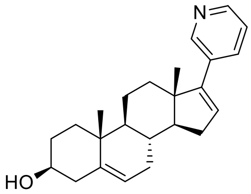	[31,32]

**sEH**	AUDA	479413-70-2	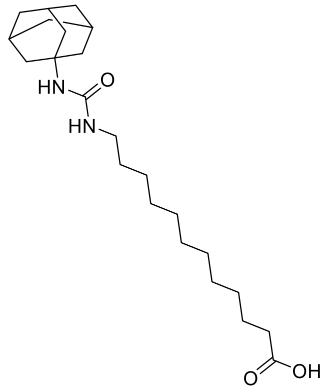	[14]

S-6. Referneces

1. Kurzemann, F. Pharmacophore modeling and virtual screening for 3β -hydroxysteroid-dehydrogenase inhibitors. Innsbruck, Leopold-Franzens Universität Innsbruck, 2016.
2. Rollinger, J.M.; Schuster, D.; Danzl, B.; Schwaiger, S.; Markt, P.; Schmidtke, M.; Gertsch, J.; Raduner, S.; Wolber, G.; Langer, T., et al. In silico target fishing for rationalized ligand discovery exemplified on constituents of *Ruta graveolens*. *Planta Med.* **2009**, *75*, 195-204, doi:10.1055/s-0028-1088397.
3. Schuster, D.; Kowalik, D.; Kirchmair, J.; Laggner, C.; Markt, P.; Aebischer-Gumy, C.; Ströhle, F.; Möller, G.; Wolber, G.; Wilckens, T., et al. Identification of chemically diverse, novel Inhibitors of 17 beta hydroxysteroid dehydrogenase type 3 and 5 pharmacophore-based virtual screening. *J. Steroid Biochem. Mol. Biol.* **2011**, *125*, 148-161.
4. Temml, V.; Garscha, U.; Romp, E.; Schubert, G.; Gerstmeier, J.; Kutil, Z.; Matuszczak, B.; Waltenberger, B.; Stuppner, H.; Werz, O., et al. Discovery of the first dual inhibitor of the 5-lipoxygenase-activating protein and soluble epoxide hydrolase using pharmacophore-based virtual screening. *Sci. Rep.* **2017**, *7*, 42751.
5. Akram, M.; Patt, M.; Kaserer, T.; Temml, V.; Waratchareeyakul, W.; Kratschmar, D.V.; Haupenthal, J.; Hartmann, R.W.; Odermatt, A.; Schuster, D. Identification of the fungicide epoxiconazole by virtual screening and biological assessment as inhibitor of human 11β-hydroxylase and aldosterone synthase. *J. Steroid Biochem. Mol. Biol.* **2019**, *192*, 105358, doi:https://doi.org/10.1016/j.jsbmb.2019.04.007.
6. Schuster, D.; Laggner, C.; Steindl, T.; Palusczak, A.; Hartmann, R.; Langer, T. Pharmacophore modeling and in silico Screening for new P450 19 (Aromatase) inhibitors. *J. Chem. Inf. Model.* **2006**, *46*, 1301-1311.
7. Hochleitner, J.; Akram, M.; Ueberall, M.; Davis, R.A.; Waltenberger, B.; Stuppner, H.; Sturm, S.; Ueberall, F.; Gostner, J.M.; Schuster, D. A combinatorial approach for the discovery of cytochrome P450 2D6 inhibitors from nature. *Sci. Rep.* **2017**, *7*, 8071, doi:10.1038/s41598-017-08404-0.
8. Vuorinen, A.; Engeli, R.T.; Leugger, S.; Bachmann, F.; Akram, M.; Atanasov, A.G.; Waltenberger, B.; Temml, V.; Stuppner, H.; Krenn, L., et al. Potential antiosteoporotic natural product lead compounds that inhibit 17β-hydroxysteroid dehydrogenase type 2. *J. Nat. Prod.* **2017**, *80*, 965-974, doi:10.1021/acs.jnatprod.6b00950.
9. Fiala, J. Pharmacophore modeling and virtual screening for 17β -hydroxysteroid dehydrogenase 7 inhibitors. Leopold-Franzens Universität Innsbruck, Innsbruck, 2017.
10. Vuorinen, A.; G., N.L.; Odermatt, A.; M., R.J.; Schuster, D. Pharmacophore model refinement for 11β-hydroxysteroid dehydrogenase inhibitors: search for modulators of intracellular glucocorticoid concentrations. *Mol. Inf.* **2013**, *33*, 15-25, doi:10.1002/minf.201300063.
11. Kratschmar, D.V.; Vuorinen, A.; Da Cunha, T.; Wolber, G.; Classen-Houben, D.; Doblhoff, O.; Schuster, D.; Odermatt, A. Characterization of activity and binding mode of glycyrrhetinic acid derivatives inhibiting 11β-hydroxysteroid dehydrogenase type 2. *The Journal of Steroid Biochemistry and Molecular Biology* **2011**, *125*, 129-142, doi:https://doi.org/10.1016/j.jsbmb.2010.12.019.
12. Brunner, F. A Pharmacophore model collection for estrogen receptor ligands using ligandscout. Leopold-Franzens Universität Innsbruck, Innsbruck, 2011.
13. Linder, T. Pharmacophore modeling and virtual screening for glucocorticoid receptor ligands: unraveling anti-inflammatory or endocrine disrupting effects of chemicals. Leopold-Franzens Universität Innsbruck, Innsbruck, 2010.

14. Waltenberger, B.; Garscha, U.; Temml, V.; Liers, J.; Werz, O.; Schuster, D.; Stuppner, H. Discovery of potent soluble Epoxide hydrolase (sEH) Inhibitors by pharmacophore-based virtual screening. *J. Chem. Inf. Model.* **2016**, *56*, 747-762, doi:10.1021/acs.jcim.5b00592.
15. Noha, S.M. Application of 3D virtual screening techniques for the identification of novel anti-inflammatory plant constituents. Leopold-Franzens Universität Innsbruck, Innsbruck, 2013.
16. Walzl, M. Molecular modeling-based analysis of 5-lipoxygenase inhibitors : common feature pharmacophore modeling and virtual screening. Leopold-Franzens Universität Innsbruck, Innsbruck, 2010.
17. Praxmarer, L. Structure- and ligand-based pharmacophore modeling for mineralocorticoid receptor ligands. Leopold-Franzens Universität Innsbruck, Innsbruck, 2011.
18. Humer, K. 3D - pharmacophore modeling for kinases involved in inflammation. Leopold-Franzens Universität Innsbruck, Innsbruck, 2009.
19. von Grafenstein, S.; Mihaly-Bison, J.; Wolber, G.; Bochkov, V.N.; Liedl, K.R.; Schuster, D. Identification of novel liver X receptor activatos by structure-based modeling. *J. Chem. Inf. Model* **2012**, *52*, 1391-1400, doi:10.1021/ci300096c.
20. Schuster, D.; Markt, P.; Grienke, U.; Mihaly-Bison, J.; Binder, M.; Noha, S.M.; Rollinger, J.M.; Stuppner, H.; Bochkov, V.N.; Wolber, G. Pharmacophore-based discovery of FXR agonists. Part I: Model development and experimental validation. *Bioorg. Med. Chem.* **2011**, *19*, 7168-7180.
21. Noha, S.M.; Jazzar, B.; Kuehnl, S.; Rollinger, J.M.; Stuppner, H.; Schaible, A.M.; Werz, O.; Wolber, G.; Schuster, D. Pharmacophore-based discovery of a novel cytosolic phospholipase A2α inhibitor. *Bioorg. Med. Chem. Lett.* **2012**, *22*, 1202-1207, doi:https://doi.org/10.1016/j.bmcl.2011.11.093.
22. Temml, V.; Kaserer, T.; Kutil, Z.; Landa, P.; Vanek, T.; Schuster, D. Pharmacophore modeling for COX-1 and -2 Inhibitors with LigandScout in comparison to Discovery Studio. *Future Med. Chem.* **2014**, *6*, 1869-1881.
23. Schuster, D.; Waltenberger, B.; Kirchmair, J.; Distinto, S.; Markt, P.; Stuppner, H.; Rollinger, J.M.; Wolber, G. Predicting cyclooxygenase inhibition by three-dimensional pharmacophore profiling. Part I: Model generation, Validation and applicability in ethnopharmacology. *Mol. Inf.* **2010**, *29*, 75-86, doi:10.1002/minf.200900071.
24. Markt, P. 3D Virtual high-throughput screening : discovery of novel lead structures and validation of the parallel screening approach with focus on targets involved in the metabolic syndrome . Kapitel 3 auch erschienen in: Journal of Computer-aided Molecular Design, 2008, online, doi: 10.1007/s10822-007-9163-6, als Artikel sobald wie möglich / Kapitel 4 auch erschienen in: Journal of Computer-aided Molecular Design, 2007, Band 10-11, Seiten 575-590. Leopold-Franzens Universität Innsbruck, Innsbruck, 2008.
25. Noha, S.M.; Fischer, K.; Koeberle, A.; Garscha, U.; Werz, O.; Schuster, D. Discovery of novel, non-acidic mPGES-1 Inhibitors by virtual Screening with multistep protocol. *Bioorg. Med. Chem.* **2015**, *23*, 4839-4845.
26. Herdlinger, S. Computer-assisted structure-activity studies on protein tyrosine phosphatase 1B (PTP1B)-inhibitors. Leopold-Franzens Universität Innsbruck, Innsbruck, 2016.
27. Noha, S.M.; Atanasov, A.G.; Schuster, D.; Markt, P.; Fakhrudin, N.; Heiss, E.H.; Schrammel, O.; Rollinger, J.M.; Stuppner, H.; Dirsch, V.M., et al. Discovery of a novel IKK-β inhibitor by ligand-based virtual screening techniques. *Bioorg. Med. Chem. Lett.* **2011**, *21*, 577-583, doi:https://doi.org/10.1016/j.bmcl.2010.10.051.

28. Rhöse, S.G. Pharmacophore modeling of antiproliferative compounds: estrogen receptor subtype-selective ligands and steroid 5α reductase inhibitors. Leopold-Franzens Universität Innsbruck, Innsbruck, 2010.
29. Grienke, U.; Kaserer, T.; Kirchweger, B.; Lambrinidis, G.; Kandel, R.T.; Foster, P.A.; Schuster, D.; Mikros, E.; Rollinger, J.M. Steroid sulfatase inhibiting lanostane triterpenes – Structure activity relationship and in silico insights. *Bioorg. Chem.* **2020**, *95*, 103495, doi:https://doi.org/10.1016/j.bioorg.2019.103495.
30. Szklarczyk, D.; Gable, A.L.; Lyon, D.; Junge, A.; Wyder, S.; Huerta-Cepas, J.; Simonovic, M.; Doncheva, N.T.; Morris, J.H.; Bork, P., et al. STRING v11: protein–protein association networks with increased coverage, supporting functional discovery in genome-wide experimental datasets. *Nucleic Acids Research* **2018**, *47*, D607-D613, doi:10.1093/nar/gky1131.
31. Udhane, S.S.; Parween, S.; Kagawa, N.; Pandey, A.V. Altered CYP19A1 and CYP3A4 activities due to mutations A115V, T142A, Q153R and P284L in the human P450 oxidoreductase. *Front. Pharmacol.* **2017**, *8*, doi:10.3389/fphar.2017.00580.
32. Samandari, E.; Kempná, P.; Nuoffer, J.-M.; Hofer, G.; E. Mullis, P.; E. Flück, C. Human adrenal corticocarcinoma NCI-H295R cells produce more androgens than NCI-H295A cells and differ in 3β-hydroxysteroid dehydrogenase type 2 and 17,20 lyase activities. *J. Endocrinol.* **2007**, *195*, 459-472, doi:10.1677/JOE-07-0166.
33. Kratschmar, D.V.; Vuorinen, A.; Da Cunha, T.; Wolber, G.; Classen-Houben, D.; Doblhoff, O.; Schuster, D.; Odermatt, A. Characterization of activity and binding mode of glycyrrhetinic acid derivatives inhibiting 11β-hydroxysteroid dehydrogenase type 2. *J. Steroid Biochem. Mol. Biol.* **2011**, *125*, 129-142, doi:https://doi.org/10.1016/j.jsbmb.2010.12.019.
34. Wetzel, M.; Marchais-Oberwinkler, S.; Perspicace, E.; Möller, G.; Adamski, J.; Hartmann, R.W. Introduction of an electron withdrawing group on the hydroxyphenylnaphthol scaffold improves the potency of 17β-hydroxysteroid dehydrogenase type 2 (17β-HSD2) inhibitors. *J. Med. Chem.* **2011**, *54*, 7547-7557, doi:10.1021/jm2008453.
35. Möller, G.; Deluca, D.; Gege, C.; Rosinus, A.; Kowalik, D.; Peters, O.; Droescher, P.; Elger, W.; Adamski, J.; Hillisch, A. Structure-based design, synthesis and in vitro characterization of potent 17β-hydroxysteroid dehydrogenase type 1 inhibitors based on 2-substitutions of estrone and D-homo-estrone. *Bioorg. Med. Chem. Lett.* **2009**, *19*, 6740-6744, doi:https://doi.org/10.1016/j.bmcl.2009.09.113.
36. Koeberle, A.; Siemoneit, U.; Bühring, U.; Northoff, H.; Laufer, S.; Albrecht, W.; Werz, O. Licofelone suppresses prostaglandin E_2 formation by interference with the inducible microsomal prostaglandin E_2 synthase-1. *J. Pharmacol. Exp. Ther.* **2008**, *326*, 975, doi:10.1124/jpet.108.139444.
37. Pandey, A.V.; Kempná, P.; Hofer, G.; Mullis, P.E.; Flück, C.E. Modulation of human CYP19A1 activity by mutant NADPH P450 oxidoreductase. *Mol. Endocrinol.* **2007**, *21*, 2579-2595, doi:10.1210/me.2007-0245.
38. Schaible, A.M.; Filosa, R.; Temml, V.; Krauth, V.; Matteis, M.; Peduto, A.; Bruno, F.; Luderer, S.; Roviezzo, F.; Di Mola, A., et al. Elucidation of the molecular mechanism and the efficacy in vivo of a novel 1,4-benzoquinone that inhibits 5-lipoxygenase. *Br. J. Pharmacol.* **2014**, *171*, 2399-2412, doi:10.1111/bph.12592.

© 2020 by the authors. Submitted for possible open access publication under the terms and conditions of the Creative Commons Attribution (CC BY) license (http://creativecommons.org/licenses/by/4.0/).
